# Infliximab Therapy Could Decrease the Risk of the Development of Thyroid Disorders in Pediatric Patients With Crohn's Disease

**DOI:** 10.3389/fendo.2020.558897

**Published:** 2020-09-15

**Authors:** Aleksandra Furtak, Anna Maria Wedrychowicz, Malgorzata Sladek, Andrzej Wedrychowicz, Krzysztof Fyderek, Jerzy Starzyk

**Affiliations:** ^1^Department of Pediatric and Adolescent Endocrinology, Chair of Pediatrics, Pediatric Institute, Medical College, Jagiellonian University in Krakow, Krakow, Poland; ^2^Department of Pediatrics, Gastroenterology and Nutrition, Pediatric Institute, Medical College, Jagiellonian University in Krakow, Krakow, Poland

**Keywords:** autoimmune thyroid diseases, Crohn's disease, Ultrasonography, Anti-TNF-alpha, pediatric patients

## Abstract

Autoimmune diseases, including autoimmune thyroid diseases (AITDs), may be associated with Crohn's disease (CD). Taking into consideration the role of tumor necrosis factor alpha (TNF-alpha) in the immune-mediated inflammation that underlies both diseases, we evaluated an ultrasound of thyroid gland in pediatric CD patients, naïve, and treated with infliximab (IFX), an anti-TNF-alpha antibody, to assess the risk for AITD and evaluated the usefulness of ultrasonography to diagnose AITD in patients with CD. Sixty-one patients with CD were enrolled in the study, including 36 patients (mean age 14.5 ± 3.5 years) treated with IFX (IFX group) for a mean of 13.9 ± 16.6 months and 25 patients (mean age 14.7 ± 2.3 years) who never received anti-TNF-alpha therapy (control group). An ultrasound examination of the thyroid gland was performed; thyroid function tests and thyroid antibodies were assessed. We found 10-times higher prevalence of decreased thyroid echogenicity in CD and IFX-naive patients compared to IFX-treated group [a significant reduction in thyroid echogenicity in 1/36 (2.8%) patients receiving IFX compared to 7/25 (28%) patients naive to biologic therapy]. The latter showed significantly lower thyroid-stimulating hormone (TSH) levels (*p* = 0.034) and higher levels of thyroid antibodies (*p* = 0.042) in comparison to control. Our data suggest the protective role of IFX therapy in the development of thyroid disorders and indicate the usefulness of thyroid ultrasound to identify the risk of probable AITD in pediatric patients with CD.

## Introduction

Crohn's disease (CD) is one of two main types of inflammatory bowel disease (IBD) that can result in progressive bowel damage and disability. The etiology of CD is multifactorial, and it is considered that chronic inflammation results from complex interactions of environmental factors, an inappropriate immune reaction against an altered microbiome in genetically susceptible individuals (1, 2). Some studies have assessed possible common genetic factors between CD and autoimmune thyroid diseases (AITDs). The role of human leukocyte antigen (HLA) genes such as PTPN22, CTLA4, and CD40 in CD patients has been extensively investigated (3). However, some studies reported that there were no significant differences in thyroid function tests {serum levels of triiodothyronine [free T3 (fT3)], thyroxine [free T4 (fT4)], and thyroid-stimulating hormone (TSH)} between CD patients and controls, or even the prevalence of thyroid dysfunction was lower in CD patients in comparison to the general population (4–6). The studies identified by the literature search indicated a 2–8% prevalence of thyroid dysfunction (hyper- or hypothyroidism) in the general population, including the populations in iodine-deficient countries (7). Snook et al. (8) reported that the prevalence of hyper- and hypothyroidism in CD amounted to 0.3 and 0.5%, respectively, similar to the control group, which was 0.7% for both. In the study of Yakut et al. (4), the prevalence of both hyper- and hypothyroidism in patients with CD as well as for both control groups was 0% (0/33) and 0% (0/66), respectively. Liu et al. (9) reported that the prevalence of hyper- and hypothyroidism in patients with CD was 0% (0/44) and 2.3% (1/44), respectively. In a study by Pooran et al. (6), the prevalence of hypothyroidism was lower in CD patients [3.8% (8/210)] than in control individuals [8.2% (17/206)], although the prevalence of hyperthyroidism was statistically similar between the groups. In a large population-based study in Canada that included 8,072 IBD patients [3,879 ulcerative colitis (UC) and 4,193 CD patients], the prevalence of Hashimoto thyroiditis (HT) was similar to that in the controls (10).

Taking into consideration the above data, a question appears: How CD therapy influences the diagnosis and clinical outcome of AITD? It is especially interesting nowadays when many patients with CD are treated with infliximab (IFX), a monoclonal anti-TNF-alpha antibody. TNF-alpha plays a role in the pathogenesis of autoimmune diseases, including thyroid diseases (11–13). Therefore, anti-TNF-alpha agents used in CD could modify concomitant autoimmune disease outcome or even may protect against them.

The diagnosis of AITD relies on the presence of circulating antibodies to thyroid antigens in blood and a typical ultrasound pattern of thyroid gland in a patient with proper clinical features and abnormal thyroid hormone levels (14). In the opinion of some experts, an ultrasonography is a more effective tool in the diagnosis and prognosis of AITD than testing for thyroid antibodies circulating in blood. According to data from a large cohort, an abnormal ultrasound pattern allows to diagnose AITD with the probability up to 95% (15–19). The lack of autoantibodies cannot exclude AITD; on the other hand, thyroid antibodies have been detected in healthy populations, also in children (20).

The primary aim of our study was the assessment of the thyroid gland morphology with ultrasonography in IFX-naive and IFX-treated pediatric CD patients. The second goal was to evaluate the usefulness of ultrasonography to assess the risk for probable AITD in pediatric patients with CD.

## Patients and Methods

We studied 61 patients with CD, treated in the Department of Pediatric, Gastroenterology and Nutrition, without any known thyroid disorder according to their medical history. Thirty-six patients were treated with IFX (IFX group), while 25 patients (control group) have never received any biologic agents. The Local Ethical Committee approved the study (No. 1072.6120.57.2019 of March 28, 2019). Parents and patients over 16 years of age signed an informed consent.

The clinical characteristic of the IFX group is presented in Table 1. All patients received biosimilar IFX, a chimeric human–mouse immunoglobulin G (IgG) monoclonal anti-TNF-alpha antibody; 29 patients received Remsima (*Biotec Services International Ltd*.), and seven patients received Flixabi [*Biogen (Denmark) Manufacturing ApS*]. In the IFX group, there were 18 girls (mean age was 14.5 ± 2.3 years) and 18 boys (mean age was 14.4 ± 4.4 years). The mean CD duration in girls was 52.6 ± 31.5 months, and the mean duration of IFX therapy was 43.8 ± 30.1 months. The mean CD duration in boys was 53.6 ± 31.7 months, and the mean duration of IFX therapy was 34 ± 19.2 months. The clinical characteristic of the control group is presented in Table 2. In the control group, there were 10 girls (mean age 13.9 ± 2.8 years) and 15 boys (mean age 15.2 ± 1.8 years). The mean CD duration in girls from the control group was 26.3 ± 32.5 months and in boys from the control group was 24.2 ± 22 months. There were no differences regarding the age and body mass index (BMI) between the groups (Table 3).

**Table 1:** The detailed characteristic of the study group—patients treated with anti-TNF alpha.

**Patient**	**Age (years)**	**Duration of Crohn disease (months)**	**Duration of anti TNF alpha therapy (months)**	**Other therapy**
1	13–14	59	59	Methotrexate
2	12–13	21	21	Mercaptopurine
3	15–16	40	29	Azathioprine, Mesalazine
4	16–17	27	21	Mercaptopurine, Mesalazine
5	11–12	91	84	Mercaptopurine
6	12–13	25	9	Azathioprine, Mesalazine
7	11–12	11	10	–
8	17–18	59	53	Mercaptopurine, Mesalazine
9	17–18	120	108	Mercaptopurine
10	12–13	65	65	Budesonide, Mesalazine
11	15–16	32	13	Azathioprine, Mesalazine
12	15–16	100	92	–
13	17–18	62	50	Azathioprine
14	11–12	26	23	Azathioprine
15	14–15	16	9	Mercaptopurine, Sulfasalazine
16	12–13	84	52	Mercaptopurine, Mesalazine
17	17–18	38	34	Azathioprine
18	13–14	72	58	Mercaptopurine, Mesalazine
19	17–18	125	34	Mesalazine
20	15–16	70	37	Mercaptopurine
21	16–17	50	23	Methotrexate, Mesalazine
22	16–17	37	35	Mercaptopurine, Mesalazine
23	16–17	36	22	Mercaptopurine, Mesalazine
24	3–4	19	9	–
25	11–12	97	71	Mesalazine
26	17–18	45	14	Azathioprine, Mesalazine
27	17–18	13	8	Mercaptopurine, Mesalazine
28	6–7	45	41	Mesalazine
29	17–18	70	69	Mercaptopurine
30	17–18	13	12	Methotrexate
31	16–17	38	38	Azathioprine, Mesalazine
32	8–9	41	37	Mercaptopurine, Sulfasalazine
33	11–12	63	41	Methylprednisolone
34	17–18	24	23	Methotrexate, Mesalazine
35	17–18	105	66	Mesalazine
36	14–15	74	32	Mesalazine
Mean data ±*SD*	14.5 ± 3.5	53.1 ± 31.2	13.9 ± 16.6	

*Patients' age is presented as a range*.

**Table 2:** The detailed characteristic of the control group—patients not treated with anti-TNF therapy.

**Patient**	**Age (years)**	**Duration of CD (months)**	**Treatment**
1	10–11	4	Azathioprine, Mesalazine
2	10–11	11	Prednisone, Mercaptopurine
3	9–10	5	Methylprednisolone, Azathioprine, Mesalazine
4	17–18	96	Methotrexate, Mesalazine, Budesonide
5	15–16	30	Methotrexate, Mesalazine
6	12–13	30	Azathioprine, Mesalazine
7	16–17	3	Budesonide, Azathioprine, Mesalazine
8	15–16	7	Azathioprine, Mesalazine
9	16–17	72	Azathioprine, Mesalazine
10	14–15	5	Budesonide, Azathioprine, Mesalazine
11	11–12	60	Azathioprine, Mesalazine
12	15–16	24	Azathioprine, Mesalazine
13	16–17	20	Methotrexate, Mesalazine
14	15–16	43	Methotrexate, Mesalazine
15	11–12	3	Methylprednisolone, Mesalazine
16	15–16	3	Methylprednisolone, Methotrexate, Mesalazine
17	16–17	3	Methotrexate, Mesalazine
18	15–16	27	Methotrexate, Mesalazine
19	15–16	36	Azathioprine, Mesalazine
20	14–15	72	Mercaptopurine, Mesalazine
21	11–12	3	Azathioprine, Mesalazine
22	16–17	36	Mesalazine
23	14–15	3	Mesalazine
24	16–17	6	Methotrexate
25	17–18	24	Azathioprine, Mesalazine
Mean data ±*SD*	14.7 ± 2.3	25.0 ± 26.1	

*Patients' age is presented as a range*.

**Table 3:** The presentation of anthropometric data and levels of thyroid tests and thyroid antibodies in patients with Crohn's disease treated (IFX group) and not treated with infliximab (control group).

**Group**	**F/M**	**Age [year]**	**Height SDS**	**BMI SDS**	**CD duration [month]**	**TSH [μIU/ml]**	**fT3 [pmol/l]**	**fT4 [pmol/l]**	**Thyroid volumne [ml]**	**aTPO [IU/ml]**	**aTG [U/ml]**	**TRAB [IU/ml]**
IFX group *N* = 36	18/18	14.5 ± 3.5	1.16 ±−0.4	1.32 ± 0.12	53.1 ± 31.2	1.31 ± 0.75	5.22 ± 0.82	13.96 ± 1.95	5.12 ± 2.15	<30	<20	0.53 ± 0.19
Control group *N* = 25	10/15	14.7 ± 2.3	−0.34 ± 1.07	−0.6 ± 1.57	25.0 ± 26.1	1.68 ± 0.79	5.33 ± 1.21	17.17 ± 2.46	5,38 ± 1.76	<30	<20	0.69 ± 0.22
*P*	0.45	0.76	0.84	0.06	<0.001	0.07	0.51	<0.001	0.63	0.97	0.99	0.03

*The data are presented as a mean ± SD*.

An ultrasound examination of the thyroid gland was performed using a Hitachi Aloka Arietta V70 in supine position with hyperextended neck using a high-frequency linear-array transducer (2–22 Hz) by the same researcher, and in doubtful cases this was followed by verification by a second specialist. Scanning was done in both transverse and longitudinal planes. Real-time imaging of thyroid lesions was performed using both gray scale and color Doppler techniques. Thyroid gland ultrasound examination included measurements of both thyroid lobes in three dimensions and thickness of thyroid isthmus. In addition, echogenicity of the thyroid parenchyma, vascularization of the gland, and presence of focal lesions were examined. Echogenicity of the thyroid gland was assessed using comparing and relationships with surrounding structures: sternocleidomastoid and strap muscles anteriorly; trachea, esophagus, and longus colli muscles posteriorly; and common carotid arteries and jugular veins bilaterally. A significant reduction of thyroid echogenicity was defined as a hypoechoic pattern of thyroid gland in comparison to submandibular gland and neck muscles. A slight reduction in thyroid echogenicity was defined as hypoechoic thyroid parenchymal pattern in comparison to submandibular gland and hyperechoic in comparison to neck muscles.

Thyroid gland function was assessed by measuring serum levels of TSH, fT3, and fT4. Moreover, anti-thyroid peroxidase antibodies (ATPOs), anti-TSH receptor antibodies (TRAbs), anti-thyroglobulin antibodies (aTGs) were measured in diagnostic process of AITD. TSH, fT3, and fT4 levels were measured using direct chemiluminescence assay (Siemens, USA). ATPO, aTG, and TRAb levels were measured using an immunochemical method with isotope label sets (Brahms, Germany). The following reference values were used: TSH 0.3–4.0 μIU/ml; fT3 3.0–8.1 pmol/l; fT4 10.0–25.0 pmol/L; ATPO <60.0 IU/ml; TRAb <1.0 IU/ml; aTG <60 U/ml.

Statistical analysis was performed using the Dell Statistica 13.1 64-bit package (StatSoft, Kraków, Poland). Variables are presented as mean with *SD*. Differences between the IFX group and the control group were determined by Student's *t*-test.

## Results

**Figure 1:**
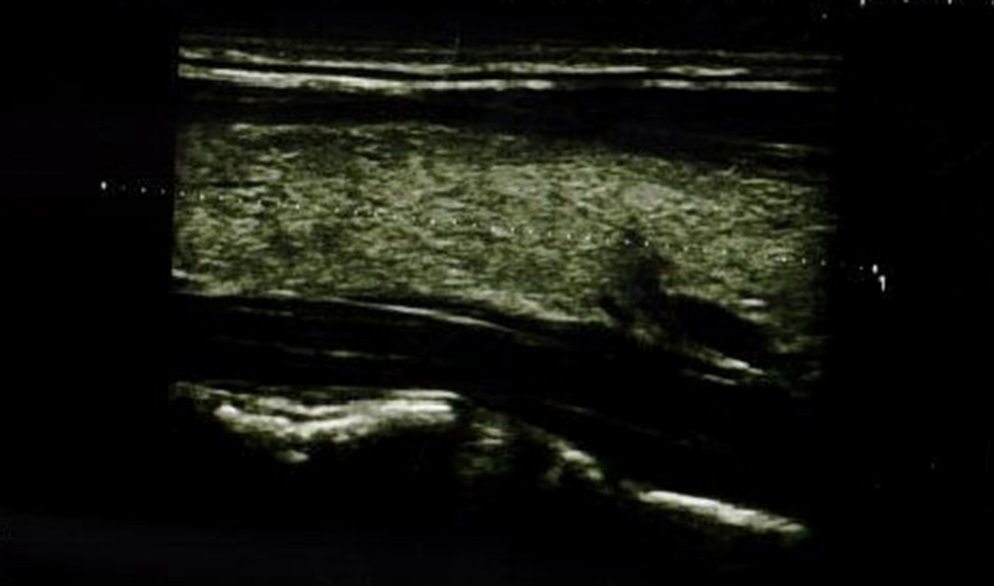
Longitudinal image of thyroid gland with heterogeneous parenchymal echo pattern.

**Figure 2:**
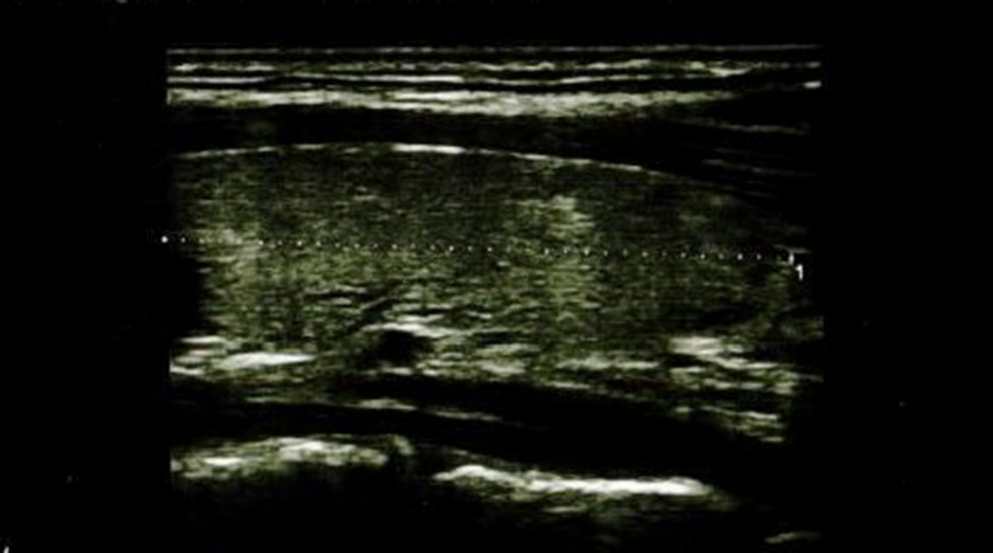
Longitudinal image of thyroid gland with slightly decreased parenchymal echo pattern.

**Figure 3:**
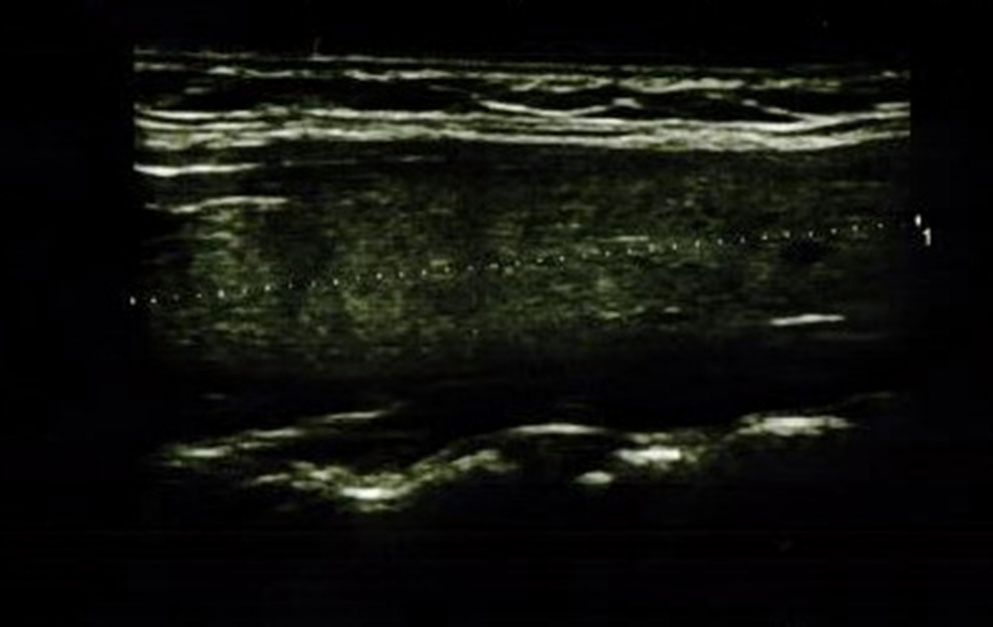
Longitudinal image of thyroid gland with significantly decreased parenchymal echo pattern.

In the IFX group, 6/36 patients (5/18 girls, 1/18 boy) had an abnormal echogenicity of thyroid gland parenchyma. In three patients, parenchymal echo pattern was heterogeneous (Figure 1); in two patients, it was slightly decreased (Figure 2), while in one case, it was decreased significantly (Figure 3). The mean CD duration in those patients was 46 ± 21.31 *SD* months (range 21–72 months). The mean duration of IFX therapy was 41 ± 17.9 *SD* months (range 21–59 months). In 8/36 patients (in two boys and five girls, including three with decreased echogenicity of the thyroid gland parenchyma), small colloid cysts located in the lower poles of the thyroid glands were found, and in one boy, two cystic solid lesions in both thyroid lobes (in left lobe 4.5 × 4.3 × 2.1 mm; in the right lobe PP 6.2 × 7.3 × 3.2 mm) were present. In this boy, ultrasound examination was repeated after 6 months, and a reduction of their dimensions and a confirmation of their cystic nature were observed. Most IFX patients (25/36) presented with a normal ultrasound pattern of thyroid gland, and all had the normal vascularization of the thyroid gland.

In the control group, an abnormal echogenicity of the thyroid gland was found in 11/25 patients (5/10 girls, 6/15 boys). In four cases, we found heterogeneous parenchymal echo pattern, and in seven, heterogeneous and significantly hypoechoic parenchymal echo pattern was visible. The mean disease duration in these patients was 26 ± 26.1 *SD* months (range 3–72 months). In 9/25 children (in four girls and five boys, including eight patients with lowered echogenicity of the thyroid parenchyma), small colloid cysts localized in the lower poles of both lobes of the thyroid glands were present. Other patients of the control group (13/25) had a normal ultrasound pattern of thyroid gland and the normal vascularization of the thyroid gland.

Thyroid function tests TSH, fT3, and fT4 were within normal ranges in both groups (Table 3). However, TSH levels were significantly lower in the IFX group compared to control. In contrary, fT4 levels were significantly higher in the control group than those in the IFX patients. No differences in fT3 levels between the two groups were found. All patients, in both groups, were negative for thyroid autoantibodies (ATPO, aTG). However, all TRAbs were negative in both groups; the titer was significantly higher in the IFX group in comparison to the control group, conversely to TSH levels. There was no difference in volumes of thyroid gland between both groups (Table 3). There was no association between abnormal thyroid ultrasound results and TRAb titer levels in the IFX group. In contrary, patients in the control group with heterogenic/hypoechoic thyroid parenchymal pattern have significantly higher TRAb levels compared to the patients with normal thyroid ultrasound (0.79 ± 0.23 vs. 0.59 ± 0.17 IU/ml, *p* = 0.042).

## Discussion

Our data could suggest the protective role of IFX therapy in the development of the thyroid disease and the usefulness of thyroid ultrasound to identify the probable risk for AITD in pediatric patients with CD.

Although the development of extraintestinal manifestations or coexistence of autoimmune disorders during the course of IBD is well-known, the coexistence of CD and thyroid diseases is still disputable (21–24). The results of our study show that the prevalence of thyroid abnormalities in CD patients is probably higher, but the outcome is different in comparison to the data from literature regarding the general population; therefore, the diagnostic criteria of thyroid disease used in the general population probably should be modified in this group of patients.

AITD is the most frequent autoimmune disease in the general population, and the most frequent form is HT (14). Most patients with HT have detectable antibodies in the blood. According to the data presented in adult patients with HT, about 81–97% of them have positive ATPO antibodies, and about 50–98% of patients have positive thyroglobulin antibodies (25). There are scarce data regarding this issue in pediatric patients with HT. About 5% of patients with HT based on clinical grounds or by ultrasound appearance have no detectable antibodies. Patients with antibody-negative HT had a milder form of hypothyroidism at the time of diagnosis. This could represent an earlier stage of the disease or simply a less aggressive form of HT (26).

Ultrasound evaluation is recommended as a screening test for patients with a high clinical risk of thyroid disease (27). The indication for thyroid ultrasonography, in spite of a diagnosis of a thyroid nodule, is to evaluate diffuse changes in thyroid parenchyma, including chronic lymphocytic thyroiditis, HT. The characteristic ultrasonography appearance of HT is focal or diffuse glandular enlargement with a coarse, heterogeneous, and hypoechoic parenchymal echo pattern. The presence of multiple discrete hypoechoic micronodules (1–6 mm in size) is strongly suggestive of chronic thyroiditis. Fine echogenic fibrous septae may produce a pseudo lobulated appearance of the parenchyma. Color Doppler may demonstrate slight to markedly increased vascularity of the thyroid parenchyma. Increased vascularity seems to be associated with hypothyroidism likely due to trophic stimulation of TSH (28). In the latter stages of HT, Doppler ultrasound findings are usually of diffuse hypovascularization and sometimes even with no detectable blood flow (29). Small atrophic gland represents end stage of HT. Occasionally, the nodular form of HT may occur, as well within a sonographic background of diffuse HT or within normal thyroid parenchyma. Moreover, ultrasonography examination may reveal the presence of perithyroidal satellite lymph nodes, especially the “Delphian” node just cephalad to the isthmus (28).

The diagnosis of AITD in patients with CD could be hindered or overlooked because of several reasons. Some signs and symptoms can be mistakenly recognized as signs and symptoms of CD or considered as adverse effects of the therapy for CD. All the more the therapy for IBD could modify the production of thyroid antibodies that are used to confirm the diagnosis of AITD and lead to false-negative results and to exclude of the disease. Wherefore in this group of patients, ultrasound examination seems to be a more useful and effective tool in the diagnosis of AITD or predict the risk in the future in patients with a normal level of thyroid hormones (30–32).

In our study, all 61 patients with CD presented with a normal thyroid function because the levels of TSH, fT3, and fT4 were normal in all cases. Moreover, all participants were negative for thyroid autoantibodies: ATPO, aTG, and TRAb. However, in 17/61 patients (27.8%), we observed a heterogeneous and hypoechoic parenchymal echo pattern of the thyroid gland. Presented data can suggest the predominance of the sensibility of thyroid ultrasound result over biochemical findings in the prognosis of the risk of probable AITD development in patients with CD. Our observations regarding the important role of ultrasonography results in the prediction of probable AITD risk in patients with IBD are in accordance with the results of studies performed in a general population.

Gutekunst et al. (19), on the basis of the results of their study performed in 92 patients with HT (aged 11–81 years), underlined the significance of ultrasound in the diagnosis of chronic lymphocytic thyroiditis. In this study, finally, chronic lymphocytic thyroiditis was confirmed by the results of cytology in 84/92 patients (91.3%). A heterogeneous parenchymal echo pattern appeared in 87/92 patients (94.6%), while antimicrosomal antibodies occurred in 80/92 (87%) patients, among which 16/80 patients (17.4%) had low titers of these antibodies (1:32–1:100) (19).

Pedersen et al. (17) indicated the value of ultrasonography in the prediction of AITD based on the analysis of 3,077 patients, referred to the study because of goiter, thyroid dysfunction, neck discomfort, and/or difficulty in swallowing. Among them, 452/552 patients had diffuse reduction in thyroid echo and were included in the study and compared with 100 control patients with a normal thyroid echogenicity. The authors of this study reported that among 110 patients with a discrete hypoechoic pattern of the thyroid gland, AITD was diagnosed finally in 87/110 patients. But among 342 participants of the study with a significant hypoechoic parenchymal pattern, AITD was diagnosed finally in 312/342. Therefore, the predictive value of a reduced thyroid echogenicity as an indicator of AITD is 79.1% for a slight reduction of thyroid echogenicity and 91.2% for a significant diffuse reduction in thyroid echogenicity. Among participants with a normal ultrasound thyroid result, only seven had finally AITD. To underline the predominance of the value of a diffuse reduction in thyroid echogenicity in the prediction of AITD, in comparison to the role of positive thyroid ATPO antibodies, the authors of the study presented that among 220 patients with a low echogenicity of the thyroid gland and confirmed AITD on the basis of biopsy results, ATPO was positive only in 162/220 patients (73.6%) (17).

In the study of Raber et al. (15) with 451 patients included, abnormal thyroid ultrasound patterns were highly indicative of autoimmune thyroiditis. Positive predictive value of significant reduction of thyroid echogenicity, understood as hypoechoic to submandibular gland and to neck muscles, for the detection of autoimmune thyroiditis was 94% with overt hypothyroidism and 96% with any degree of hypothyroidism. Positive predictive value of the slight reduction in thyroid echogenicity, understood as hypoechoic to submandibular gland, hyperechoic to neck muscles, is 85 and 87%, respectively (15).

Rago et al. (16) presented thyroid ultrasonography as a tool for detecting thyroid autoimmune diseases and predicting thyroid dysfunction in apparently healthy subjects. Among 482 healthy subjects, living in a borderline iodine-sufficient urban area, 41 had thyroid hypoechogenicity, and in this group, 11 had an abnormal thyroid function (seven with positive and four with negative thyroid autoantibodies). None of the 429 participants of the study with normal thyroid echostructure had thyroid dysfunction, although 12 had positive thyroid autoantibodies. Although positive TPO and/or aTG was more frequent (24/482, 5%) in subjects with thyroid dysfunction (7/11) than in those who remained euthyroid during the study (17/471, χ^2^ = 69.66, *p* < 0.0001), thyroid hypoechogenicity had a higher sensitivity than the positivity of thyroid autoantibody tests (100 vs. 63.3%) for diagnosing or predicting thyroid dysfunction (16).

The results of our study not only indicate that ultrasound assessment could be a sensitive tool in detecting thyroid abnormality but also suggest that therapy with IFX can modify the clinical course. To our knowledge, this is the first such observation. The only study reported to date regarding the influence of anti-TNF-alpha therapy on the thyroid gland function did not present data on ultrasonography (33). The aim of this cited study was to investigate for the first time the thyroid function in patients with IBD and the potential effect of anti-TNF-alpha therapy. Forty-one patients with IBD, without any known thyroid disorder, were evaluated. Eighteen patients were on anti-TNF-alpha therapy for more than 1 year. From the second group, 12 of 23 patients on conventional therapy (azathioprine plus mesalazine) were put on anti-TNF-alpha and studied 6 months later. Anti-TNF-alpha-treated patients presented with significantly lower fT4 levels, but still within normal ranges, and no differences in TSH and T3 levels. The percentage of patients with positive thyroid antibodies was lower in the anti-TNF-alpha group, but not significantly. After 6 months of treatment with anti-TNF-alpha, fT4 levels were found to be reduced, while no changes in TSH and T3 levels and thyroid autoantibodies were noted. The advantage of this study comparing to ours is the long-term observation. However, the results, based only on biochemical results, seem to be in agreement with our results based on ultrasound of thyroid glands.

We found a significant reduction in thyroid echogenicity in 1/36 (2.8%) patients receiving IFX compared to 7/25 patients (28%) naive to biologic therapy, although the duration time of CD in the IFX group had been longer in comparison to controls. Therefore, this 10-times higher prevalence of significant reduction in thyroid echogenicity in CD patients without anti-TNF-alpha therapy expressly suggests the preventive role of IFX in the probable development of AITD. Moreover, IFX patients have significantly lower levels of TSH without differences of thyroid volumes and higher thyroid antibody levels in comparison to the control naive group, although both groups did not differ regarding BMI and age. Our observations together with the knowledge from literature about the role of TNF-alpha in the pathogenesis of both AITD and IBD suggest that thyroid ultrasound could be a useful tool in the identification of CD pediatric patients at risk for AITD.

The advantage of our study is the novel observation of the possible preventive role of IFX therapy in the development of thyroid abnormalities probably preceding AITD. On the basis of the presented data, we propose thyroid ultrasound as a useful tool in the identification of the risk for thyroid disease in pediatric patients. All the more thyroid ultrasound is easily accessible, non-invasive, and cost-effective. The main disadvantage of the ultrasonography is that this method is operator dependent. For this reason, in our study, all participants were examined by one physician and verified by a second one, always the same two persons (34). In differential diagnosis of AITD, other diffuse thyroid diseases should be taken into consideration, multinodular goiter, de-Quervain's subacute thyroiditis, and Graves disease, because the sonographic features of these processes may be similar. However, these conditions have different biochemical profiles and clinical presentations. Therefore, always, ultrasound findings should be viewed in relation to clinical and biochemical status of the patient. The most dangerous, but possible, diagnostic pitfall is that diffuse infiltrative vascular thyroid carcinoma like papillary or follicular carcinoma may be mistaken for AITD. Ultrasonography features that suggest malignancy include irregular or nodular enlargement of the thyroid gland, local invasion, and nodal metastases. Sometimes, these features are not visible at once, and such cases require observation with repeated ultrasonography examination (35). Long-term observation is indicated in each case with an abnormal thyroid picture in ultrasonography. In AITD, abnormal ultrasound pictures never normalize and remain for the rest of the patient's life. Moreover, HT is associated with an increased risk of thyroid malignancies like follicular or papillary carcinoma and lymphoma (36). All the more there are data that in patients with inflammatory bowel diseases, focal lesions relating to tumors of the thyroid gland are more common than in the control group (37).

The weaknesses of the presented study are an uneven distribution of girls and boys in both groups and a lack of long-term observation. Female sex has a higher risk of thyroid diseases, so it could influence the results in both groups. However, in our control group with fewer girls than boys, we found more abnormal patterns of thyroid glands just in the boys in comparison to the girls. It would be very interesting how will be the further outcome of thyroid function and its morphology in the presented patients. Therefore, we plan to follow up our patients and repeat the study after 12 months with renewed assessment of thyroid antibodies in both groups.

Summarized, we propose thyroid ultrasound for use as an easily accessible, non-invasive tool to identify the risk of thyroid abnormalities probably preceding AITD in pediatric patients with CD. Because CD treatment especially with TNF blockers could modulate the AITD presentations, the thyroid ultrasonography should be considered before starting IFX therapy, and a long-term follow-up may be necessary in case of abnormal thyroid findings.

## Data Availability Statement

The datasets presented in this article are not readily available because, the data are the property of the patients. Requests to access the datasets should be directed to Jagiellonian University—Medical College.

## Ethics Statement

The studies involving human participants were reviewed and approved by Jagiellonian University Ethical Committee approved the study (No. 1072.6120.57.2019 of March 28, 2019). Written informed consent to participate in this study was provided by the participants' legal guardian/next of kin.

## Author Contributions

AMW and JS contributed to the concept. AMW and AF contributed to the design, contributed to data collection or processing, contributed to analysis or interpretation, literature search, and contributed to writing. AF, AMW, MS, AW, and KF contributed to the medical and surgical practices. All authors contributed to the final version of the manuscript.

## Conflict of Interest

The authors declare that the research was conducted in the absence of any commercial or financial relationships that could be construed as a potential conflict of interest.

## References

[1] Neumann MG. Immune dysfunction in inflammatory bowel disease. Transl Res. (2007) 149:173–86. 10.1016/j.trsl.2006.11.009 17383591

[2] Cho JH Brant SR. Recent insights into the genetics of inflammatory bowel disease. Gastroenterology. (2011) 140:1704–12. 10.1053/j.gastro.2011.02.046 21530736 PMC4947143

[3] Marinò M Latrofa F Menconi F Chiovato L Vitti P. Role of genetic and non-genetic factors in the etiology of Graves' disease. J Endocrinol Invest. (2015) 38:283–94. 10.1007/s40618-014-0214-2 25421156

[4] Yakut M Üstün Y Kabacan G Soykan I Thyroid disorders in patients with inflammatory bowel diseases. Int J Clin Med. (2011) 2:89–92. 10.4236/ijcm.2011.22018

[5] Tunc B Filik L Ulker A Demirbag A Sahin B. Subclinical thyroid disorders and inflammatory bowel disease. Romanian J Gastroenterol. (2005) 14:98–9. 15800702

[6] Pooran N Singh P Bank S. Crohn's disease and risk of fracture: does thyroid disease play a role? World J Gastroenterol. (2003) 9:615–8. 10.3748/wjg.v9.i3.615 12632531 PMC4621595

[7] Casella G De Marco E Antonelli E Daperno M Baldini V Signorini S. The prevalence of hyper- and hypothyroidism in patients with ulcerative colitis. J Crohn's Colitis. (2008) 2:327–30. 10.1016/j.crohns.2008.09.001 21172232

[8] Snook JA de Silva HJ Jewell DP. The association of autoimmune disorders with inflammatory Bowel disease. Quart J Med. (1989) 72:835–40. 2616728

[9] Liu S Ren J Zhao Y Han G Hong Z Yan D. Nonthyroidal illness syndrome: is it far away from Crohn's disease? J Clin Gastroenterol. (2013) 47:153–9. 10.1097/MCG.0b013e318254ea8a 22874844

[10] Bernstein CN Wajda A Blanchard JF. The clustering of other chronic inflammatory diseases in inflammatory bowel disease: a population-based study. Gastroenterology. (2005) 129:827–36. 10.1053/j.gastro.2005.06.021 16143122

[11] Choudhury P Chakraborty S Saha A Mazumder S Association of serum TNF-Alpha with thyroid parameters: a hospital based study. Int J Res Rev. (2019) 6:30–2.

[12] Polińska B Matowicka-Karna J Kemona H. The cytokines in inflammatory bowel disease. Postepy Hig Med Dosw. (2009) 63:389–94. 19724079

[13] Aust G Heuer M Laue S Lehmann I Hofmann A Heldin NE. Expression of tumour necrosis factor-alpha (TNF-alpha) mRNA and protein in pathological thyroid tissue and carcinoma cell lines. Clin Exp Immunol. (1996) 105:148–54. 10.1046/j.1365-2249.1996.d01-726.x 8697623 PMC2200483

[14] Caturegli P De Remigis A Rose NR. Hashimoto thyroiditis: clinical and diagnostic criteria. Autoimmun Rev. (2014) 13:391–7. 10.1016/j.autrev.2014.01.007 24434360

[15] Raber W Gessl A Nowotny P Vierhapper H. Thyroid ultrasound vs. antithyroid peroxidase antibody determination: a cohort study of four hundred fifty-one subjects. Thyroid. (2002) 12:725–31. 10.1089/105072502760258712 12225642

[16] Rago T Chiovato L Grasso L Pinchera A Vitti P. Thyroid ultrasonography as a tool for detecting thyroid autoimmune diseases and predicting thyroid dysfunction in apparently healthy subjects. J Endocrinol Invest. (2001) 24:763–9. 10.1007/BF03343925 11765045

[17] Pedersen OM Aardal NP Larssen TB Varhaug JE Myking O Vik-Mo H. The value of ultrasonography in predicting autoimmune thyroid disease. Thyroid. (2000) 10:251–9. 10.1089/thy.2000.10.251 10779140

[18] Rubello D Gasparoni P Rota G Borsato N Zanco P Chierichetti F. Functional meaning of scintigraphic and echographic patterns, and of circulating anti-peroxidase antibodies in asymptomatic chronic thyroiditis. Quart J Nucl Med. (1996) 40:359–64. 9050341

[19] Gutekunst R Hafermann W Mansky T Scriba PC. Ultrasonography related to clinical and laboratory findings in lymphocytic thyroiditis. Acta Endocrinol. (1989) 121:129–35. 10.1530/acta.0.1210129 2662693

[20] Taubner K Schubert G Pulzer F Pfaeffle R Körner A Dietz A. Serum concentrations of anti-thyroid peroxidase and anti-thyroglobulin antibodies in children and adolescents without apparent thyroid disorders. Clin Biochem. (2014) 47:3–7. 10.1016/j.clinbiochem.2013.09.017 24103918

[21] Cesarini M Angelucci E Rivera M Pica R Paoluzi P Vernia P. Thyroid disorders and inflammatory bowel diseases: retrospective evaluation of 909 patients from an Italian Referral Center. Inflam Bowel Dis. (2010) 16:186–7. 10.1002/ibd.20964 19462424

[22] Gimondo P Mirk P Pizzi C Messina G Gimondo S Iafrancesco G. Clinico-ultrasonographic assessment of the thyroid volume and function in chronic enteritis and colitis: preliminary data. Radiol Medica. (1996) 92:257–60. 8975312

[23] Halling ML Kjeldsen J Knudsen T Nielsen J Hansen LK. Patients with inflammatory bowel disease have increased risk of autoimmune and inflammatory diseases. World J Gastroenterol. (2017) 23:6137–46. 10.3748/wjg.v23.i33.6137 28970729 PMC5597505

[24] Kappelman MD Galanko JA Porter CQ Sandler RS. Association of paediatric inflammatory bowel disease with other immune-mediated diseases. Arch Dis Child. (2011) 96:1042–6. 10.1136/archdischild-2011-300633 21903597

[25] Nishihara E Amino N Kudo T Ito M Fukata S Nishikawa M. Comparison of thyroglobulin and thyroid peroxidase antibodies measured by five different kits in autoimmune thyroid diseases. Endocrine J. (2017) 64:955–61. 10.1507/endocrj.EJ17-0164 28768936

[26] Rotondi M de Martinis L Coperchini F Pignatti P Pirali B Ghilotti S. Serum negative autoimmune thyroiditis displays a milder clinical picture compared with classic Hashimoto's thyroiditis. Eur J Endocrinol. (2014) 171:31–6. 10.1530/EJE-14-0147 24743395

[27] Gharib H Papini E Garber JR Duick DS Harrell RM Hegedüs L. American Association of Clinical Endocrinologists, American College of Endocrinology, and Associazione Medici Endocrinologi medical guidelines for clinical practice for the diagnosis and management of thyroid nodules−2016 update. Endocrine Practice. (2016) 22:622–39. 10.4158/EP161208.GL 27167915

[28] Chaudhary V Bano S Thyroid ultrasound. Indian J Endocrinol Metab. (2013) 7:219–27. 10.4103/2230-8210.109667 PMC3683194 23776892

[29] Takahashi MS Pedro HMM Chammas MC Ultrasound evaluation of thyroiditis: a review. J Otolaryngol Res. (2019) 2:127.

[30] Dayan CM Daniels GH. Chronic autoimmune thyroiditis. N Engl J Med. (1996) 335:99–107. 10.1056/NEJM199607113350206 8649497

[31] Marcocci C Vitti P Cetani F Catalano F Concetti R Pinchera A. Thyroid ultrasonography helps to identify patients with diffuse lymphocytic thyroiditis who are prone to develop hypothyroidism. J Clin Endocrinol Metab. (1991) 72:209–13. 10.1210/jcem-72-1-209 1986019

[32] Hayashi N Tamaki N Konishi J Yonekura Y Senda M Kasagi K Sonography of Hashimoto's thyroiditis. J Clin Ultrasound. (1986) 14:123–6. 10.1002/jcu.1870140208 3081583

[33] Paschou SA Palioura E Kothonas F Myroforidis A Loi V Poulou A. The effect of anti-TNF therapy on thyroid function in patients with inflammatory bowel disease. Endocr J. (2018) 65:1121–5. 10.1507/endocrj.EJ18-0243 30135331

[34] Russ G Bonnema SJ Erdogan MF Durante C Ngu R Leenhardt L. European thyroid association guidelines for ultrasound malignancy risk stratification of thyroid nodules in adults: the EU-TIRADS. Eur Thyroid J. (2017) 6:225–37. 10.1159/000478927 29167761 PMC5652895

[35] Januś D Wójcik M Taczanowska A Sołtysiak P Wedrychowicz A Roztoczyńska D. Follow-up of parenchymal changes in the thyroid gland with diffuse autoimmune thyroiditis in children prior to the development of papillary thyroid carcinoma. J Endocrinol Invest. (2019) 42:261–70. 10.1007/s40618-018-0909-x 29872995 PMC6394764

[36] Anderson L Middleton WD Teefey SA Reading CC Langer JE Desser T. Hashimoto thyroiditis: part 2, sonographic analysis of bening and malignant nodules in patients with diffuse Hashimoto thyroiditis. AJR Am J Roentgenol. (2010) 195:216–22. 10.2214/AJR.09.3680 20566819

[37] Neubauer K Wozniak-Stolarska B. Ultrasonographic assessment of the thyroid gland structure in inflammatory bowel disease patients. Adv Clin Exp Med. (2012) 21:43–6. 23214298

